# Optimized production and characterization of ulvan from *Ulva Lactuca* with *in vitro* biological activities

**DOI:** 10.1038/s41598-026-44503-7

**Published:** 2026-04-02

**Authors:** Amira M. Abu-Resha, Mostafa M. El-Sheekh, Ghada W. Abou-El-Souod, Hanaa Morsi, Mofida Makhlof

**Affiliations:** 1 Botany and Microbiology Department, Faculty of Science, Menoufia University, Shibin Elkoum, Egypt; 2 Botany Department, Faculty of Science, Tanta University, Tanta, 31527 Egypt; 3 Botany and Microbiology Department, Faculty of Science, Damanhour University, Damanhour, Egypt

**Keywords:** *Ulva lactauca*, Ulvan, Thermal stability, Pancreatic cancer, Antioxidant activity, Hepatitis A virus (HAV), Biochemistry, Biotechnology, Cancer, Drug discovery, Microbiology, Plant sciences

## Abstract

Bioactive sulfated polysaccharides, including ulvan, known for their broad therapeutic uses, are abundant in green algae. In this study, *Ulva lactuca* ulvan was produced under optimized conditions involving temperature, pH, extraction time, and solvent concentration adjustments. The extracted ulvan (*U. lactuca* ulvan, ULU) was identified using analytical methods such as Fourier Transform Infrared Spectroscopy (FTIR), Thermogravimetric Analysis (TGA), X-ray Diffraction (XRD), Scanning Electron Microscopy (SEM), High-Performance Liquid Chromatography (HPLC), and Energy-Dispersive X-ray Spectroscopy (EDX) to confirm its compositional stability. A maximum yield of 23.33 ± 0.28% was achieved, with rhamnose as the main sugar, accounting for 14.10 µg/g of dried weight. Ulvan’s biological activities are likely influenced by its sulfate content, which measured 35.35 ± 0.25%. The bioactivity of ULU was tested for anticancer, antioxidant, and antiviral effects. ULU showed significant anticancer activity against pancreatic cancer cells (Panc-1). At a dose of 1000 µg/ml, ULU achieved an inhibition rate of 86.15%, with an IC_50_ of 123.51 ± 10.95 µg/ml, demonstrating substantial effectiveness in inhibiting pancreatic cancer cell growth. In antioxidant tests, ULU exhibited moderate free radical scavenging activity against DPPH, with a maximum inhibition rate of 88.31% ±2.64% at 1000 µg/ml and an IC_50_ of 263.73 ± 9.41 µg/ml. These results highlight the benefits of improved extraction and optimized conditions, indicating that ULU has potential as a natural antioxidant, though with modest efficacy compared to ascorbic acid. ULU also demonstrated limited antiviral activity, with an inhibition rate of 40.25 ± 2.61% against Hepatitis A virus (HAV) at 50 µg/ml, as measured by the MTT assay. The cytotoxicity test showed a CC₅₀ value of 230.53 ± 2.09 µg/ml, indicating a moderate safety margin. Despite its limited antiviral effectiveness, further research into combining ULU with other drugs or exploring synergistic effects could enhance its therapeutic potential.

## Introduction

Saltwater algae (seaweeds) have considerable potential as a resource for next-generation biorefineries^1^. According to González Fernández et al.^2^, the distinctive algal extracts render it an increasingly promising alternative for diverse applications. Macroalgae could be classified into green, red, and brown categories^3^. Chlorophyta includes species that are called green algae^4^.

Some green algae species inhabit freshwater and saltwater ecosystems. Marine macroalgae from the Chlorophyta phylum mainly include the Ulvophyceae class^5^. *Ulva lactuca*, a species of green seaweed, constitutes a significant biomass. It is distinguished by a notably unique biological composition, particularly its elevated concentration of cell wall polysaccharides^6^. Sulfated polysaccharides (SPs) are negatively charged polysaccharides characterized by sulfate groups within their carbohydrate backbone, which may be naturally occurring or synthetically produced.

Ulvan (Ulv) is predominant in *Ulva* algae walls^7^, which constitutes 9–36% of *Ulva* dry weight^8^. Research indicated that Ulv primarily consists of different monosaccharides with different degrees of sulfation, exhibiting variability according to harvest season, species, growth environment, cultivation methods, and extraction techniques, and the interest in Ulv is due to its effective biological activity^9,10^.

Cancer, a significant health threat, primarily results from abnormalities in cell division. Disruptions may arise from genetic, chemical, and environmental factors^11^. Pancreatic cancer (PDAC) has become the third most lethal solid malignancy in the United States, following lung and colorectal cancer, and is projected to become the second-leading cause of cancer-related deaths in the United States by 2030^12^. Exposure to chemical agents, including heavy metals, particularly at sufficiently high doses, can induce severe toxic effects such as cardiotoxicity, hepatotoxicity, neurotoxicity, nephrotoxicity, and potentially fatal hematopoietic toxicity, which may contribute to carcinogenic processes^13^. Therefore, novel antitumor compounds that exhibit minimal toxicity should be discovered^14^. Consequently, numerous researchers have concentrated on identifying novel anticarcinogenic compounds derived from algae and plants^15^.

Impairs the transmission and control of redox signals has the potential to cause molecular damage; this is known as oxidative stress. It has a role in the etiology of many diseases and the aging process^16^. Sulfated polysaccharides produced from algae, particularly ulvan, offer antioxidant and free-radical scavenging properties that may protect living things from oxidative harm^17,18^.

Viral infections are prevalent and impose considerable strain on healthcare systems globally. The availability of specific antiviral drugs for numerous viral infections is limited, resulting in symptomatic treatment and systemic supportive therapy being the main approaches^14^. There is an urgent requirement for innovative and efficient agents^19^. Investigations into natural antiviral agents exhibiting carbohydrate characteristics have underscored the significance of sulfated polysaccharides, such as ulvan, which exhibit notable antiviral properties^20^.

Herein, *U. lactuca* ulvan (ULU) was extracted, optimized, characterized, and assessed for its biological activities, such as anticancer effects against Panc-1 (pancreatic cancer), along with DPPH free radical scavenging assay, and potential of anti-Hepatitis A virus (HAV). Based on the findings, this study will be essential in exploring Ulvan’s potential utility as a natural molecule in cancer treatment, antioxidant therapy, and perhaps antiviral applications, laying the groundwork for future research and development in the biomedical and pharmaceutical industries.

## Experimental section

### *Ulva lactuca* sampling

*Ulva lactuca* was collected by Prof. Fekry Ashour Mourad, affiliated with the National Oceanography and Fisheries Institute in Egypt, during May 2022, specifically from the Gulf Suez coast of Egypt. Algal samples were collected from a non-protected public beach, where no permission or licence was required, and where no commercial use or genetic resource export was involved. Morphological and taxonomic identification were then conducted using the methods outlined by Aleem^21,22^, accompanied by a microscopic examination as described by Lipkin^23^, and subsequently verified through the Algae Base website^24,25^. After collection, samples underwent multiple H_2_O washes to exclude any associated biota or sand, etc. Following this, *U. lactuca* were rinsed again with fresh H_2_O to exclude salts, then 72 h in a shaded area before being oven-dried at 60 °C for 3 h using a Memmert oven from Germany; milling occurred via Brown Mill coffee grinder from Berlin, Germany, to obtain *U. lactuca* powder (ULp) that was stored for future experimental use. The voucher specimen (*Ulva lactuca*—Herb. Nasr-1Gr7-5–2022) has been deposited at the herbarium of late Professor Abdel-Halim Nasr at the Botany and Microbiology Department, Faculty of Science, Alexandria University, Egypt, with the help of Prof. Mohamed Saad Abdelkareem.

### (ULU) extraction optimization

ULp de-pigmentation involved using 100 mL hexane for a day, accompanied by 3000 rpm shaking to eliminate non-polar impurities. The ULp was subsequently filtered. Then, it was submerged in 120 mL of 95% ethanol for another whole day, with moderate agitation, to eliminate soluble contaminants^26^. Following a three-hour exposure to a vacuum at 60 °C, the residue underwent drying.

In the subsequent phase of the experiment, 30 g of dried seaweed powder underwent a series of hot water extraction processes aimed at isolating ulvan (sulfated heteropolysaccharides) under a range of conditions, temperatures (40 °C, 60 °C, 80 °C, 100 °C, 120 °C, 140 °C, and 160 °C), seaweed-to-water ratios (1/10, 1/20, 1/30, 1/40, 1/50, and 1/60 w/v), a variety of pH levels (1.5, 3, 4.5, 6, and 7.5), and a range of extraction durations (20, 35, 50, 65, 80, 95, 110, and 125 min). Following filtration, the liquid portion underwent centrifugation at 6708× g for a quarter-hour, after which the supernatant was dialyzed for 2 days at 4 °C against H_2_O to eliminate minor contaminants. The obtained liquid portion volume was reduced via rotary evaporator and precipitated with four volumes of 100% ethanol at − 20 °C. Following 48 h, the precipitate was centrifuged and dried under vacuum at 60 °C.

Final ulvan extract yield (UY) measured as follows:

UY % = (ULU wt./ULp wt.) × 100^27^.

### Chemical analysis of (ULU)

#### Sulfate content (SC)

According to Torres et al.^28^, sulfate content was estimated, which corroborates its consistency with traditional procedures^29^. Gelatin reagent (GR) and BaCl_2_ were initially made by mixing 75 mg gelatin in 25 mL H_2_O, then incubation occur at 80 °C for 10 min with vigorous mixing, then the addition of 250 mg barium chloride took place, finally kept at 4 °C. By using 96-well clear polystyrene microplate (WPM), 140 µL of 0.5 mol/L HCl were added in each well followed by the addition of 20 µL (non-hydrolyzed and hydrolyzed ULU) and 0.5 mol/L of the negative control HCl. After mixing with 40 µL GR in each well for 20 min, absorbance at 405 nm was determined via a Sunrise microplate reader device (TECAN, Woburn, Inc, USA, MA), subsequently SC was determined as follow:

SC = {(µH)− (µN) }± p σH² + σN².

Where σH and µH represent the standard deviation and mean of SC for hydrolyzed ULU, respectively and σN and µN represent those for non-hydrolyzed ULU, respectively.

#### Total Sugar Composition (TSC) and Total Protein Composition (TPC)

TSC and TPC were calculated in order to determine the Purity degree of ULU according to phenol–sulfuric acid method^30^ and the Lowry method^31^, respectively.

#### Moisture and ash content

Via oven heating ULU for 24 h at 103 °C, % dry weight was measured^32^. Ash content was calculated gravimetrically by drying 70 mg ULU at 550 °C for 14 h^8^.

#### Elemental analysis (EA)

C, N, H, and S in ULU were performed with a Vario Micro Cube(VMC), Germany Elementer (GE).

#### Uronic acid (UA)

UA was calculated via the meta-hydroxy diphenyl method^33^. In this procedure, 0.4 mL of 1 mg/mL ULU was applied to 2.4 mL of conc H_2_SO_4_, the mixture was heated for 30 min up to 100 °C with 120 mM sodium tetraborate, after cooling, 150 µL of m-hydroxydiphenyl reagent was applied with 15 min incubation, then ab was determined at a range (400 to 700 nm).

### (ULU) characterization

#### HPLC

Monosaccharide composition of ULU was determined via HPLC (Agilent, USA, California) after a process of acid hydrolysis using different reference monosugars^34^.

#### FTIR

ULU active chemical groups were analyzed using an FTIR spectrometer (Bruker, Germany, ALPHA), which measured within the 4000–400 cm⁻¹ range, using a resolution of 4.0 cm⁻¹ and averaged over 128 scans^35^.

#### XRD

The crystallinity of Ulvan were detected using the Bruker D2 Phaser X-ray diffractometer, which is equipped with Cu Kα radiation (λ = 1.5412 Å) and operates at 30 mA and 40 kV over a 2θ range of 5° to 85°.

#### TGA

Thermogravimetric analysis (TGA) tests were conducted on a Netzsch (DSC) 204 through a stable flow of nitrogen. An amount of 1.71 mg ULU was progressively heated at 20 °C to 1000 °C per min., with recording the thermo-gram^8^.

#### (SEM & EDX) analysis

High Resolution Scan Electron Microscope {(HRSEM), Jeol, JSM-IT 200, Japan} using a 15 kV accelerating voltage was applied to study ULU surface morphology^36,37^. While ULU element composition was determine via the same device with an energy-dispersive X-ray spectrometer (EDX)^38^.

#### ULU cytotoxicity

Vero Cells (VCs) (derived from the kidney of an African green monkey) with the Panc-1 cell line supplied by the American Type Culture Collection (ATCC), Rockville, M, culturing in Dulbecco’s modified Eagle’s medium (DMEM) was done with a day of incubation in 37 °C at conc 5 × 10⁴ cells/well, ULU was then added to the 96-WP, with six wells containing media or 0.5% DMSO, used as controls and after a whole day, cell viability was measured and viability % was determine as follow^39^.

Viability%= {(ODs/ODc)} × 100%, where ODs and ODc represent the optical density of ULU and the control, respectively. Inhibitory conc (IC_50_), defined as the concentration inducing 50% toxicity in intact cells, was measured using the software GraphPad (CA, San Diego, USA).

### DPPH free radical scavenging activity of (ULU)

Different concentrations of ULU were used at concentrations ranging from 2 to 1000 µg/mL, from each conc 4 µL was added to 3 mL of a DPPH solution prepared at 0.004% (w/v). Using the UV spec (Spectronic 1201, Milton Roy), absorbance was measured at 515 nm using ascorbic acid as reference, inhibition percentage % (IP) was measured as described below:

IP = {(AS - AC)/AS} x 100], where AS and AC represent the ab of test and control, respectively^40^.

### Antiviral activity of (ULU) against Hepatitis A virus(HAV)

HAV (HM175) strain (ATCC, VR:1402) was cultured and tested in relation to vero cells (VCs) via MTT method^41–43^; after viral and VCs propagation with conc 2 × 10^5^ cells/100 L, ULU was added for each well, OD at 590 nm was then measured with an ELISA reader (SunRise, TECAN, Inc., USA), to viral determine inhibition % (VI) with the evaluation of IC_50_ as follow^39^.

VI=(A − N)/(B − N) ˣ100%.

In which A, N, and B corresponded to the OD of ULU in virus-infected cells, the OD of the virus control and the OD of the cell control, respectively.

### Statistical analysis

Statistical analysis was performed using SPSS software, version 9.4.1 (CA, San Diego, USA). Comparisons were made using one-way ANOVA, followed by Tukey’s post hoc test, with significance set at *p* < 0.05.

## Results and discussion

**Fig. 1:**
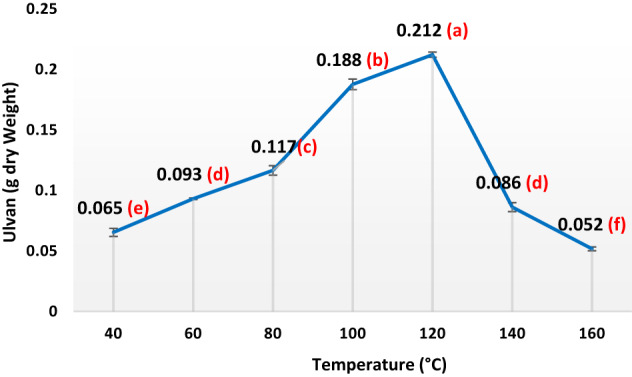
Effect of different temperature degrees on ulvan production from *Ulva lactauca* using a one factor at a time (OFAT) approach with (Time:30 min., pH:4, alga: water ratio,1:20 kept constant). Yield per 1 g of dry alga increased from 0.065 g at 40 ℃ to 0.212 g at 120 ℃. *The letters (**a**,** b**, and **c**) indicate a significance level of *P* < 0.05. *Values sharing the same letter are not significantly different. *Note: All experimental data representing the effect of optimized conditions were statistically analyzed using SPSS software (version 9.4.1, San Diego, CA, USA). One-way ANOVA followed by Tukey’s post hoc test was applied, and differences were considered significant at *p* < 0.05.

**Fig. 2:**
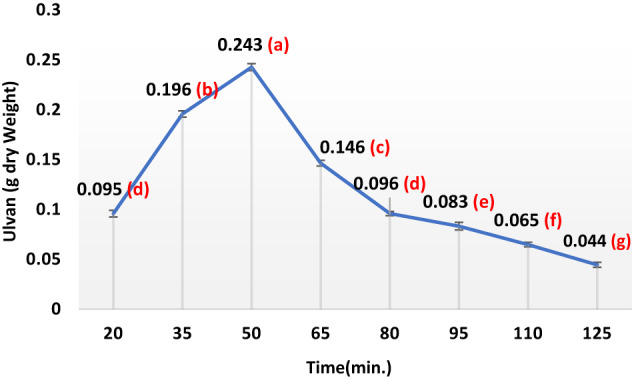
Impact of varying extraction times on ulvan yield from *Ulva lactauca* using a one factor at a time (OFAT) approach with (Temperature 120 ℃, pH:4, alga: water ratio, 1:20 kept constant). Yield per 1 g of dry alga increased from 0.095 at 20 min to 0.243 at 50 min.

**Fig. 3:**
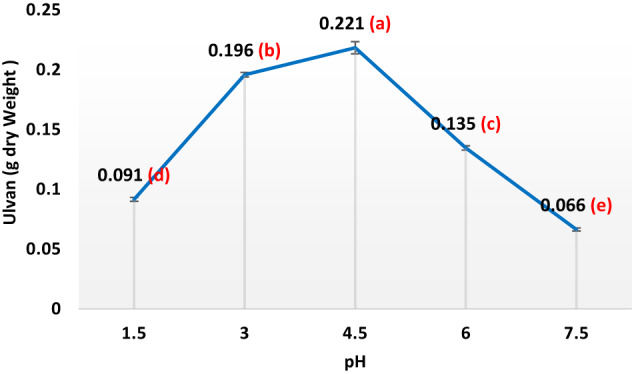
Effect of varying pH levels on ulvan production in *Ulva lactauca* using a one factor at a time (OFAT) approach with (Temperature 120 ℃, Time:50 min, alga: water ratio, 1:20 kept constant). Yield per 1 g of dry alga increased from 0.091 at pH 1.5 to 0.221 at pH 4.5.

**Fig. 4:**
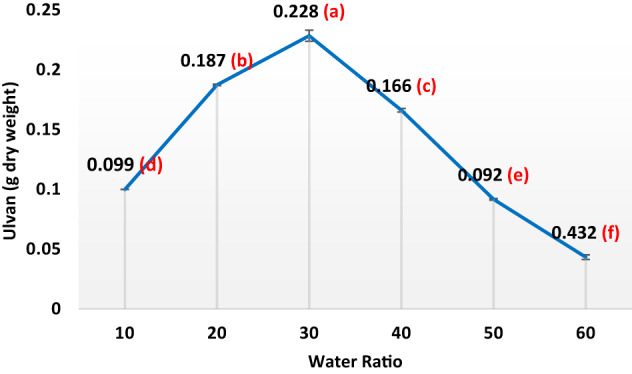
Impact of varying water volumes on ulvan production in *Ulva lactauca* using a one factor at a time (OFAT) approach with (Temperature 120 ℃, Time:50 min, pH 4.5 kept constant).Yield per 1 g of dry alga increased from 0.099 at 1:10 to 0.228 at 1:30, the final optimized yield.

**Fig. 5:**
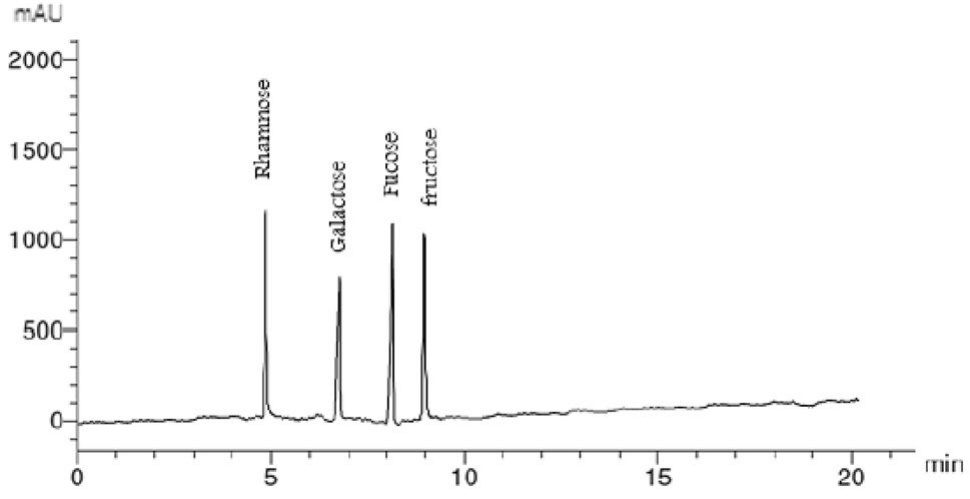
HPLC Chromograph of Ulvan from *Ulva lactauca*.

Ulvans were produced using hot-water extraction followed by ethanol precipitation, as described by Binsuwaidan et al.^44^. Hot water extraction produced the maximum yield when compared to other studies^45,46^. The extraction conditions were optimized at 120 °C for 50 min, with a pH of 4.5 and a 1:30 ratio of algal powder to water (Figs. 1, 2, 3 and 4). This process yielded 23.33 ± 0.28% from 30 g of algal powder (Table 1). The yield was twice that reported by Binsuwaidan et al.^44^ (11.20 ± 0.32%) and exceeded those reported by Ponce et al.^47^ for *Ulva ohnoi* (14.84%), Chen et al.^48^ (17.8 ± 0.6%), and Klongklaewad et al.^49^ (15.2%). Table 1 summarizes the chemical composition and elemental analysis of the extracted ulvan (ULU), while Table 2 presents a comparison of *Ulva* species regarding extraction and purification methods, conditions, yield, and sulfate content^44–53^. Although advanced techniques such as microwave-assisted extraction (MAE) and ultrasound-assisted extraction (UAE) can achieve higher ulvan yields, they require specialized equipment, precise control of energy, and may cause partial degradation or alteration of sensitive compounds^54,55^. In contrast, our hot water extraction method offers a simpler, cost-effective, and reproducible approach suitable for large-scale production. The concentration of uronic acid was 5.19 µg/while the sulfate content was 35.35 ± 0.25%, considerably better than sulfate amounts reported by Ibrahim et al.^56^ (19.72%) and Binsuwaidan et al.^44^ (14.95%), which is responsible for the majority of the activities of ulvans^57,58^. Furthermore, TSC was 42.74 ± 0.32%, surpassing that of Ibrahim et al.^56^ (24.27%) and Binsuwaidan et al.^44^ (33.66%). The elevated sugar content (42.74 **±** 0.32%) and reduced protein content (5.15 ± 0.05%) may be associated with heightened photosynthetic activity in May, which promotes growth and development^59,60^. The extract exhibited a moisture content of 56.4 ±0.35%, indicative of the hydrophilic and hygroscopic characteristics of ulvan polysaccharides^61^. In addition, the ash content was 39.001 **±**0.26%, indicating a correlation between high ash content and raised sulfate levels, as shown by Costa et al.^62^, Olasehinde et al.^63^, and Ibrahim et al.^56^. The HPLC analysis (Fig. 5) delineated the monosaccharide composition of ULU, which includes rhamnose (14.10 µg/g dry weight), fucose (11.04 µg/g dry weight), fructose (10.35 µg/g dry weight), and galactose (7.41 µg/g dry weight), with rhamnose as the predominant sugar. Rhamnose is a significant indicator of ulvan^64^, with a small amount of galactose^10^.

**Table 1:** Proximate chemical analysis and monosaccharide composition of Ulvan from *Ulva lactauca*.

Parameter measured	Value (%) ± SD
Yield	23.33 **±** 0.28
Dry weight	7.02 **±** 0.02
Fresh weight	10.07 **±** 0.12
Moisture content	56.4 **±** 0.35
Chemical Constituents	Dry weight (%) ± SD
Total ash	39.001 **±** 0.26
Total protein content	5.15 **±** 0.05
Total carbohydrate content	42.74 **±** 0.32
Sulphate concentration	35.35 **±** 0.25
Moisture content	7.98 **±** 0.34
Monosaccharide composition	(µg/gm)
Rhamnose	14.10
Fucose	11.04
Fructose	10.35
Galactose	7.41
Uronic acids	5.19
Elemental analysis	(%)
Carbon content	34.05
Nitrogen content	10.61
Hydrogen content	2.94
Sulfur content	0.87

**Table 2:** Comparison of *Ulva* species: Extraction and purification methods, conditions, yield, and sulfate content in relation to the present study.

Ulva species	Extraction method	Purification method	Solvent type	Temperature (℃)	Time	Yield (%)	Sulphate content	Reference
*Ulva lactuca* (Gulf of Suez, Egypt)	Hot water extraction	Ethanol precipitation	Water	80 ℃	2 h	11.203%	14.95%	^44^
*Ulva rotundata* *(Pleubian*,* Côte d’Armor*,* France)*	Acidic extraction (0.05 M Hot dilute HCl)	Neutralization + Freeze drying	HCl	85 ℃	60 min	21.5%	13.3%	^45^
*Ulva lactuca* (Swedish west coast)	Hot water extraction	Ethanol wash + α-amylase + Proteinase K + dialysis	Water	90 ℃	3 h	11%	6.01%(reported as sulfur content)	^46^
*Ulva ohnoi*) (*outlet channel of the IFAPA El Toruño facilities) *	Hot water extraction	pH precipitation + ultrafiltration	water	80 ℃	2 h	14.84%	20.24%	^47^
*Ulva pertusa* Kjellm (the western Pacific coast)	Hot water extraction with 0.05 M sodium oxalate	Ethanol precipitation	Water	90 ^◦^C	3 h	17.8%	13.2%	^48^
*U. intestinalis* (earthen ponds on a private seaweed farm in Pattani Province)	Hot water extraction	Freeze-drying	Water	110 ^◦^C	90 min	15.2%	13.75%	^49^
*Ulva lactuca* (Ujung Genteng, Indonesia*)*	Ultrasound-assisted Extraction	Ethanol/Isopropyl + alcohol precipitation	Water	80 °C	2 h	26.32%	Confirmd by FTIR, (1213 and 1050 cm⁻¹)	^50^
*Ulva prolifera* (China)	Microwave-assisted hydrothermal	Ethanol precipitation +lyophilization	HCl solution (0.01 M)	120 °C	15 min	36.38%	7.66%	^51^
*U. meridionalis* (Japan)	Microwave-assisted extraction (2.45 GHz)	Ethanol precipitation + lyophilization	Water	160 °C	5 min heating + 5 min holding	43.8%	Detected by FTIR (1262 and 1056 cm^–1^)	^52^
*Ulva fenestrata)(Portugal)*	Enzyme-assisted extraction (Cellulysin)	Ultrafiltration (10 kDa), ethanol precipitation, freeze-drying	0.1 M sodium acetate buffer, pH 5	40 °C	20 h	14.1%	Detected by FTIR (1260 cm^–1^)	^53^
*Ulva lactauca* (Gulf Suez coast of Egypt)	Hot water extraction after de-pigmentation (hexane + ethanol)	Filtration + Centrifugation + Dialysis + Ethanol precipitation + Vacuum drying	Water	120 ^◦^C	50 min	23.33%	35.35%	This study

Note: Our hot water extraction yielded 23.33% ulvan, higher than other conventional hot water methods (11–17.8%) for similar conditions. Although modern techniques like microwave- or ultrasound-assisted extraction achieve higher yields (26.32–43.8%), they require specialized equipment, higher energy input, and may cause partial degradation or altered composition of ulvan, limiting large-scale and consistent production. Our method uses moderate heating and chemicals, making it cost-effective, reproducible, and suitable for large-scale production.

### FTIR

**Fig. 6:**
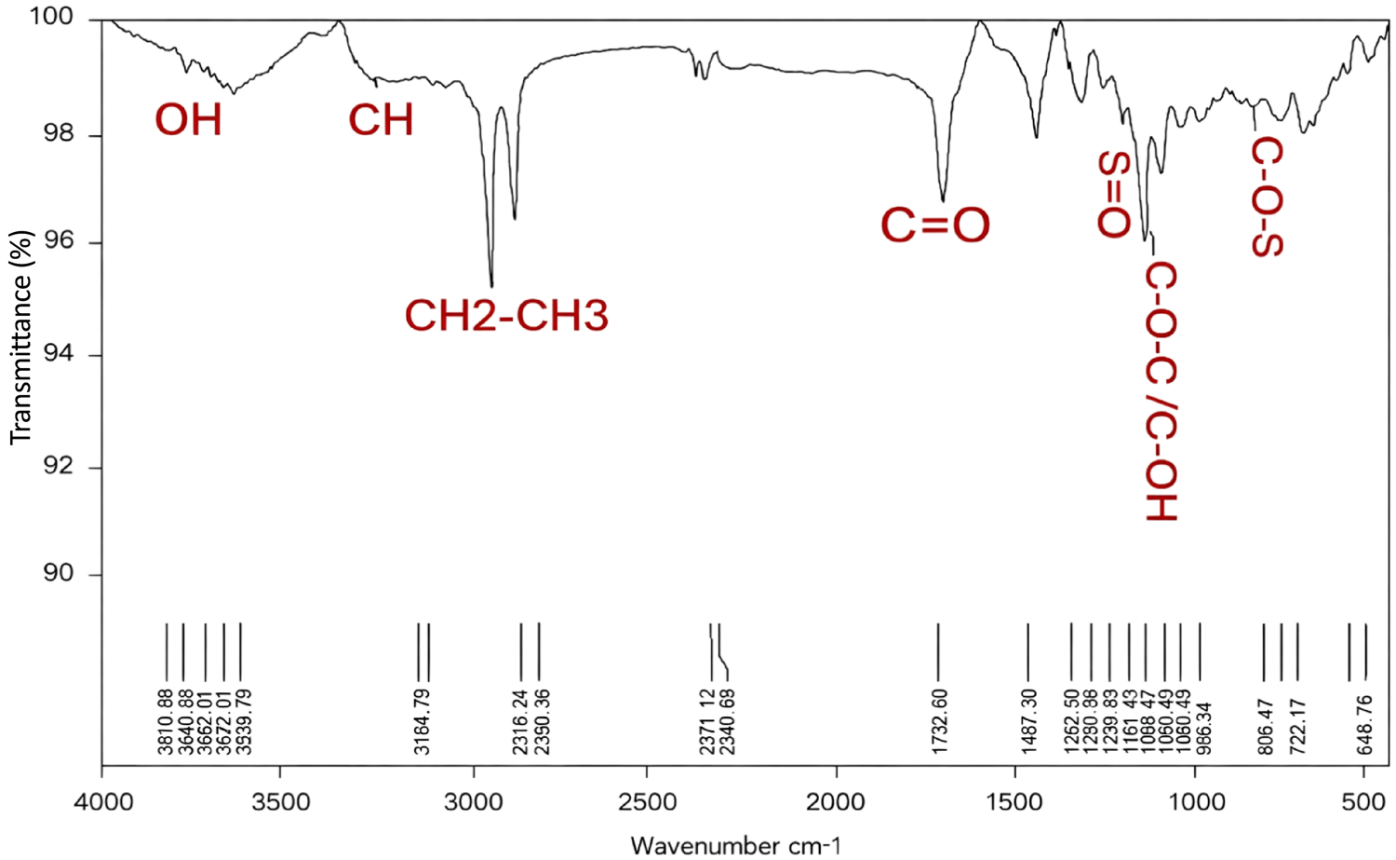
FT-IR spectra of ulvan extracted from *Ulva lactauca.*

The FTIR spectrum of ULU (Fig. 6) demonstrates multiple functional groups that signify its chemical structure, with purity similar to that of other *Ulva*members, according to Quemener et al.^65^; Tian et al.^66^; Mhatre et al.^67^; Olasehinde et al.^63^; Ibrahim et al.^56^; Binsuwaidan et al.^44^ and Maray et al.^68^.

The distinctive hydroxyl groups (OH) present in polysaccharides are responsible for the wide absorption band in the 3810.80–3629.79 cm⁻¹ area. That means the Ulvan structure does include sugar-based functional groups. A peak at 3184.79 cm⁻¹ indicates C-H vibrations, particularly from CH₂ groups. The observed peaks at 2918.24 and 2850.36 cm⁻¹ correspond to the C-H vibrations, which are characteristic of hydrocarbon chains (CH₂ and CH₃) found in the sugar backbone of polysaccharides, thereby reinforcing the organic composition of Ulvan^69^. Atmospheric interferences or CO₂ may be present in the region 2371.12–2340.69 cm⁻¹. It is a typical feature of FTIR spectra and has no impact on the analysis of Ulvan’s fundamental functional groups. The absorption at 1732.80 cm⁻¹ corresponds to C = O stretching vibrations, signifying carbonyl groups (C = O), often associated with uronic acids, a constituent of the Ulvan structure, hence corroborating the existence of polymeric characteristics in the sample^70^. The peaks at 1467.30–1342.50 cm⁻¹ indicate C-H bending and O-H deformation vibrations linked to aliphatic and hydroxyl groups within the polysaccharide^66^. The strong absorption in the range of 1280.88–1161.47 cm⁻¹ is due to S = O groups, indicating the presence of sulfate groups, a characteristic feature of Ulvan^56^, which contributes to its biological activities^58^. The range 1098.62–986.34 cm⁻¹ is related to C-O-C and C-OH restricting glycosidic bonds and hydroxyl groups in the polysaccharide sugar backbone^66^. The absorption within the range of 805.07–648.17 cm⁻¹ indicates C-O-S vibrations, hence substantiating the existence of sulfate esters, a crucial structural component in sulfated polysaccharides such as Ulvan. The peak at 455.76 cm⁻¹ is ascribed to low-frequency vibrations, presumably associated with the skeletal modes of the sugar backbone, offering more structural insights.

### XRD

**Fig. 7:**
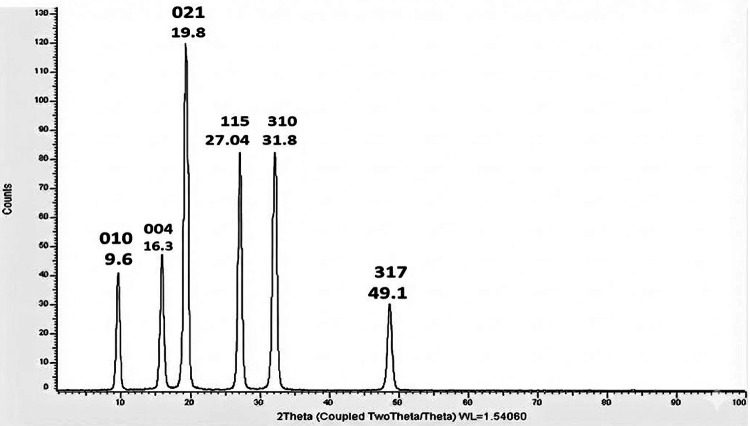
X-ray diffraction (XRD) analysis of ulvan extracted from *Ulva lactauca.*

Figure 7shows that ULU has a semi-crystalline nature characterized by prominent peaks in accordance with Binsuwaidan et al.^44^.

The dominant peak at 2θ = 19.8° correlates to 021 plane, exhibiting a substantial level of structural order characteristic of semi-crystalline polysaccharides. Further peaks identified at 2θ values of 9.6° (010), 16.3° (004), 27.04° (115), 31.8° (310), and 49.1° (317) substantiate the ordered structure inside the ulvan matrix, enhancing its rigidity and stability. Although ulvan and similar polysaccharides are often considered amorphous^71^, this pattern demonstrates harmony between amorphous and semi-crystalline areas.

Olasehinde et al.^63^ proposed that repeated aldobiouronic units may explain its crystalline area, whereas Barakat et al.^71^ suggested that its amorphous region could be due to its heterogeneous chemical makeup. In biomaterials, the semi-crystalline areas improve thermal stability and structural integrity; in medicinal formulations, the amorphous parts improve bioavailability by increasing solubility and flexibility.

### TGA

**Fig. 8:**
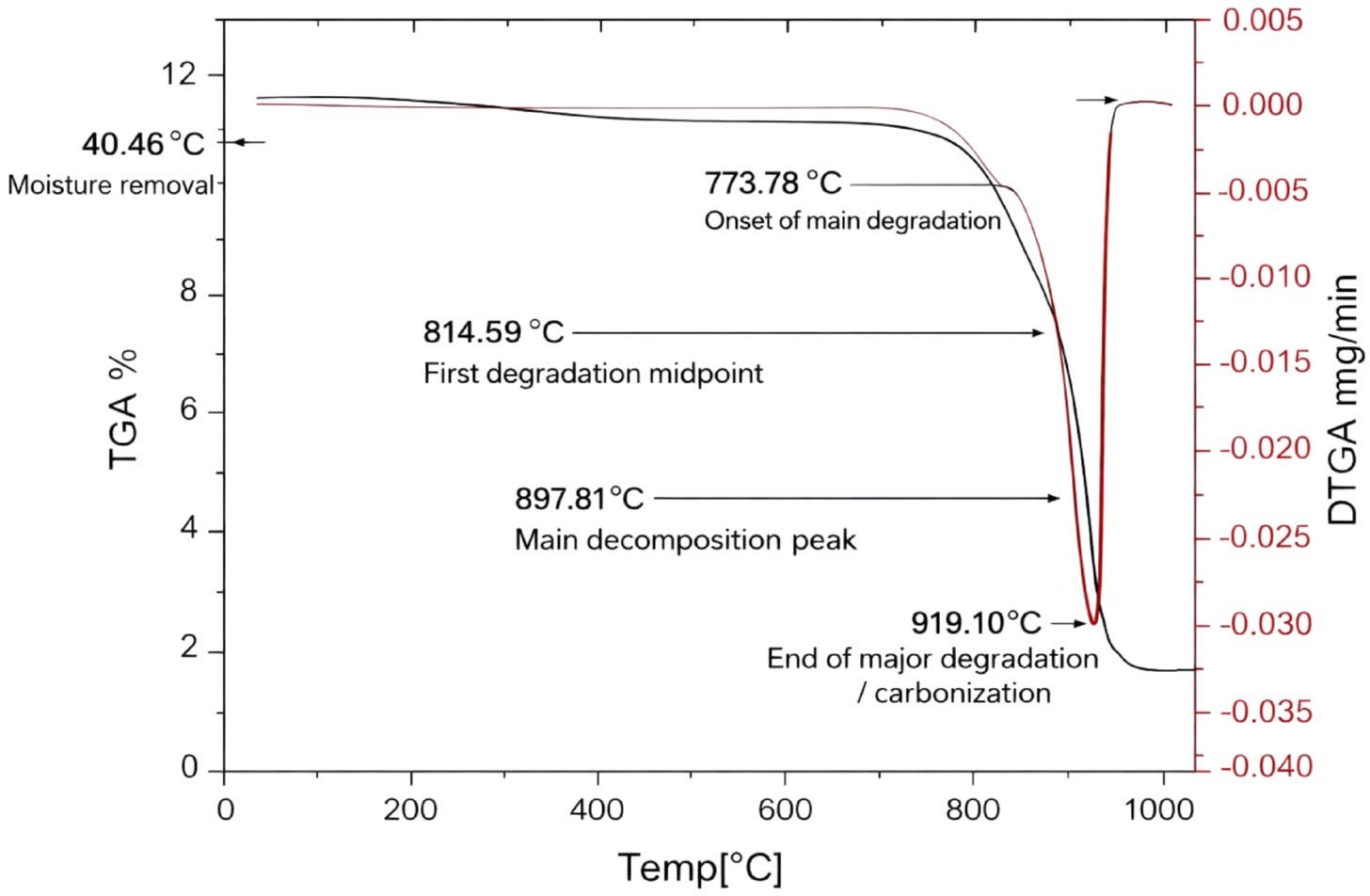
TGA of Ulvan from *Ulva lactauca*.

The thermal stability and decomposition characteristics of the ULU (Fig. 8) have been examined using thermogravimetric analysis (TGA). The findings are similar to those reported by Gruskiene et al.^72^. The TGA curve revealed the weight loss pattern over a temperature range from 40 °C to 1000 °C, emphasizing key stages of thermal breakdown. Weight loss started between 40.46 °C and 860.73 °C, with a notable beginning at 773.78 °C and ending at 864.86 °C. The midpoint of this phase was 814.59 °C, with a total weight decrease of 3.304 mg, equal to a 23.419% reduction. This phase likely indicates the removal of residual moisture and volatile organic compounds, suggesting that the original components of Ulvan stay stable up to about 770 °C, after which significant thermal disintegration begins. The main decomposition occurred between 860.73 °C and 994.93 °C, starting at 876.15 °C and ending at 919.10 °C, with a midpoint at 897.81 °C. During this phase, substantial weight loss was observed, with a reduction of 8.275 mg, or 58.656% of the total weight. This large weight loss suggests the breakdown of the core polysaccharide structure and its conversion into volatile products. The notable degradation at high temperatures highlights the durability of Ulvan’s molecular framework against decomposition, indicating the temperature at which its thermal stability is most tested. The results show that Ulvan maintains significant thermal stability up to about 770 °C, after which gradual decomposition occurs. The initiation of decomposition at higher temperatures indicates that Ulvan polysaccharides have inherent thermal resistance, likely due to their complex structure with sulfated polysaccharide chains.

### ULU SEM and EDX

**Fig. 9:**
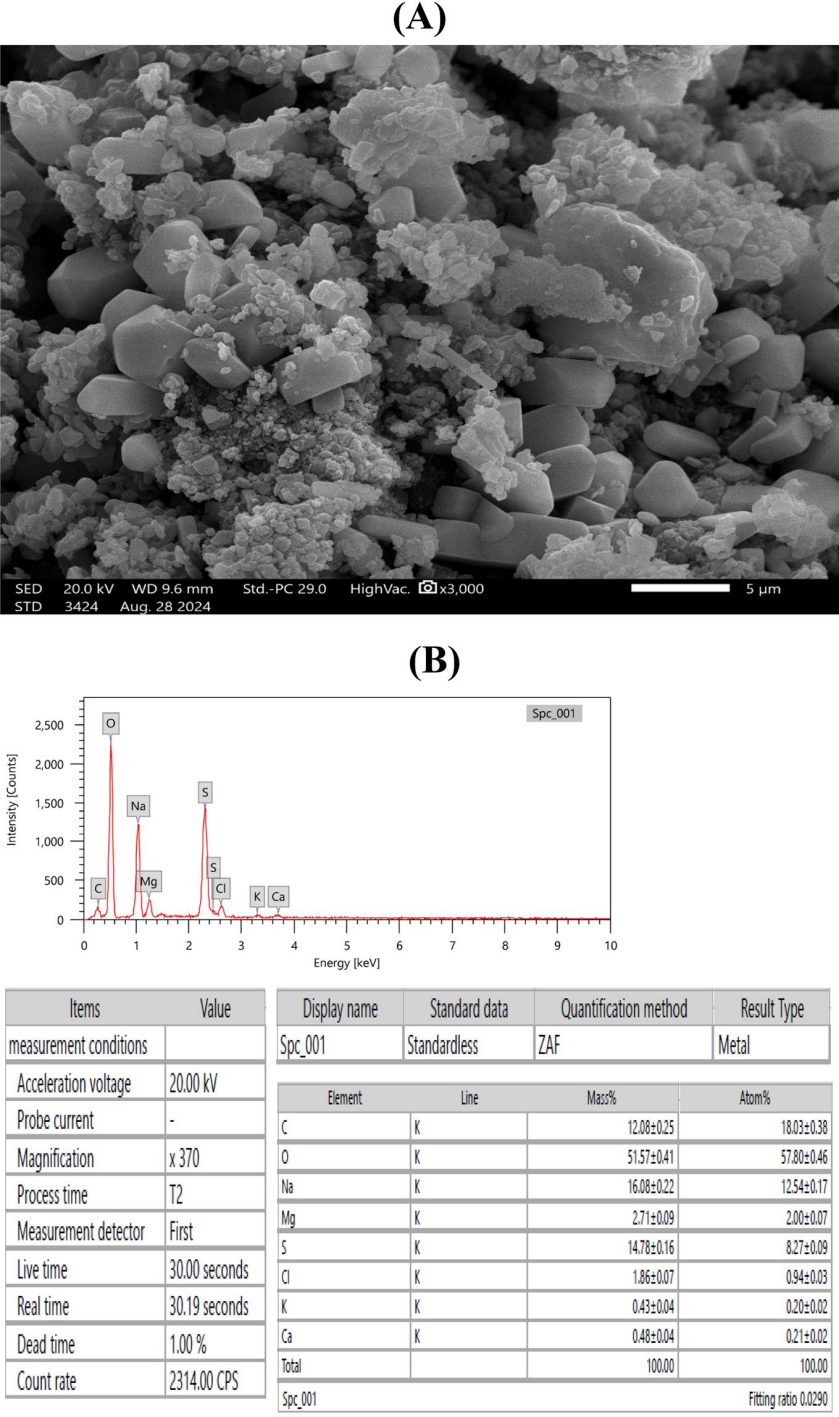
SEM micrographs (**A**), (scale bar 5 μm) and corresponding EDX elemental of ulvan (**B**) extracted from *Ulva lactuca*, showing various surface morphologies. The SEM images reveal semi-crystalline structures, which are closely correlated with the distribution of elements such as sulfur (S), sodium (Na), and magnesium (Mg) detected by EDX, suggesting that these elemental compositions may contribute to the observed crystalline-like features rather than originating solely from the polymeric chains of ulvan.

According to Binsuwaidan et al.^44^, ULU has a semi-crystalline structure, is not smooth, and resembles *Ulva lactauca* in its irregular forms (Fig. 9 [A]). Further EDX examination (Fig. 9 [B]) revealed a variety of elements, with oxygen (O) accounting for 51.57%, carbon (C) for 12.08%, sulfur (S) for 14.78%, magnesium (Mg) for 2.71%, potassium (K) for 0.43%, calcium (Ca) for 0.48%, sodium (Na) for 16.08%, and chloride (Cl) for 1.86%.

### Cytotoxicity of ULU

**Fig. 10:**
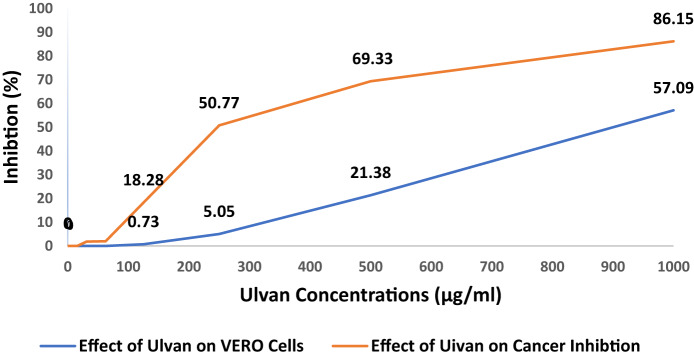
Cytotoxic effect of ulvan from *Ulva lactauca* on Panc-1 and Vero cell lines.

The cytotoxicity illustrated in Fig. 10 reveals a dose-dependent inhibitory activity. At lower concentrations (2–62.5 µg/ml), the compound exhibited minimal or no significant cytotoxicity, with viability percentages surpassing 98%. However, at concentrations of 125 µg/ml and higher, a noticeable decrease in cell viability was observed, indicating a significant cytotoxic effect. The maximum inhibition (86.15%) occurred at 1000 µg/ml, resulting in a viability of 13.85%. The determined IC_50_value of 123.51 ± 10.95 µg/ml suggests that ULU has considerable cytotoxicity towards Panc-1 cells; this agrees with Maray et al.^68^, who reported nearly similar results of natural polysaccharides tested against lung cancer cells. The close alignment in IC_50_ values indicates that ULU shares comparable potency with previously studied polysaccharides in targeting cancer cells. Previous studies stated that the heteropolysaccharide ulvan extracted from *Ulva* showed significant antitumor growth activity^73^. As shown in Fig. 10, increasing concentrations of ulvan led to significant inhibition of cancer cell proliferation while exhibiting minimal effects on healthy cells. This suggests that ulvan possesses selective cytotoxicity towards cancer cells, highlighting its potential as a therapeutic agent.

### DPPH scavenging

Many studies demonstrate the antioxidant properties of ulvan^48,74,75^. Results in Table 3 suggest a concentration-dependent increase in scavenging activity, substantiating the compound’s antioxidant capability. At the lowest evaluated concentration (2 µg/mL), the scavenging activity was negligible (0.98% ±0.13). As the concentration increased, the activity markedly elevated, reaching 88.31% ±2.64 at the maximum concentration (1000 µg/mL). The IC_50_value, indicating the concentration necessary to scavenge 50% DPPH radicals, was established at 263.73 ± 9.41 µg/mL. Conversely, Maray et al.^68^ extracted ulvan from *Ulva lactuca* with similar techniques but indicated little antioxidant activity, with an IC_50_ value significantly above 263.73 µg/mL. This divergence underscores the heterogeneity in antioxidant activity, which may be associated with changes in algae species or the extraction methods utilized in contrast^46^. Antioxidant action relies on the sulfate content^76^. Although seasonal variation was not assessed in this study, recent research has demonstrated seasonal changes in the composition and antioxidant potential of *Ulva lacinulata*^77^. Such findings indicate that environmental and seasonal factors may influence ulvan’s antioxidant activity.

**Table 3:** DPPH scavenging activity of Ulvan from *Ulva lactauca*.

Sample conc. (µg/ml)	DPPH scavenging % (±S.D)
0	0 ± 0
2	0.98 ± 0.13
3.9	1.74 ± 0.09
7.8	3.85 ± 0.18
15.6	5.63 ± 0.32
31.25	10.84 ± 0.49
62.5	32.71 ± 0.53
125	51.20 ± 0.71
250	62.57 ± 0.63
500	80.43 ± 1.79
1000	88.31 ± 2.64

The present work reveals that ULU has considerable antioxidant activity, with a notably lower IC_50_ value, signifying higher effectiveness than previously documented. While the current study did not explore chemical modifications or combinations with other bioactive compounds, such approaches could be investigated in future research to assess their potential impact on ulvan’s antioxidant capacity for broader medicinal and commercial applications.

### ULU Virucidal

Koenighofer et al.^78^ indicate that ulvan significantly inhibits several virus strains. These chemicals inhibit viral entry by disrupting its binding with the target cell. ULU Virucidal effect could originate from ULU binding with viruses or receptors located on its surfaces^79^. Table 4 revealed moderate antiviral activity (40.25 ± 2.61%) at conc 50 µg/ml. EC₅₀ value for Ulvan was 60.95 ± 0.43 µg/ml, whereas the cytotoxic concentration (CC₅₀) was 230.53 ± 2.09 µg/ml, resulting in a Selectivity Index (SI) of 3.7.

**Table 4:** Antiviral activity of Ulvan from *Ulva lactauca*.

Sample name	MNCC (µg/ml)	Antiviral effect on HAV (%)	Antiviral effect on HAV(Qualitative)	Antiviral Efficiency		
				EC_50_	CC_50_	SI
*U. lactauca* ulvan	50	40.25 ± 2.61	++	60.95 ± 0.43	230.53 ± 2.09	3.7
Reference drug (Amantadine)	100	84.95 ± 6.53	++++	5.67 ± 0.71	302.85 ± 4.93	53.41

Where (-): No antiviral activity.

(+): Weak antiviral activity (1-<25%).

(++): Moderate antiviral activity (25-<50%).

(+++): Good antiviral activity (50-<75%).

(++++): Excellent antiviral activity (75–100%).

The results indicate moderate antiviral activity of ulvan. Previous studies have shown that combining ulvan with other polysaccharides, such as fucoidan, can modulate antiviral effects, although synergistic activity is not guaranteed, and ulvan may antagonize certain effects^80^. Additionally, recent studies have highlighted that extraction conditions and algal morphology can significantly influence the chemical, thermal, and molecular properties of ulvan^81–83^, providing context for understanding the physicochemical profile of the polysaccharide observed in this study.

## Conclusion

The Ulvan polysaccharide derived from *Ulva lactuca* (ULU) has significant anticancer activities, particularly at elevated amounts, highlighting its role as a promising viable treatment option; also, it may serve as a biosubstitute for chemical antioxidants. Nonetheless, its antiviral is also taken into consideration; recommended studies should focus on augmenting its virucidal effectiveness by optimizing its amounts, investigating synergizing medicines, or any other promising technique to boost its activities.

## Data Availability

All data generated or analyzed during this study are included in the current article, and any further requests for data should be from the corresponding author.
